# Advances in Vertebral Augmentation Systems for Osteoporotic Vertebral Compression Fractures

**DOI:** 10.1155/2020/3947368

**Published:** 2020-12-07

**Authors:** Yufeng Long, Weihong Yi, Dazhi Yang

**Affiliations:** ^1^Health Science Center, Shenzhen University, Shenzhen 518071, Guangdong Province, China; ^2^Department of Spine Surgery, The 6th Affiliated Hospital of Shenzhen University Health Science Center, Shenzhen 518052, Guangdong Province, China

## Abstract

Osteoporotic vertebral compression fracture (OVCF) is a common cause of pain and disability and is steadily increasing due to the growth of the elderly population. To date, percutaneous vertebroplasty (PVP) and percutaneous kyphoplasty (PKP) are almost universally accepted as appropriate vertebral augmentation procedures for OVCFs. There are many advantages of vertebral augmentation, such as short surgical time, performance under local anaesthesia, and rapid pain relief. However, there are certain issues regarding the utilization of these vertebral augmentations, such as loss of vertebral height, cement leakage, and adjacent vertebral refracture. Hence, the treatment for OVCF has changed in recent years. Satisfactory clinical results have been obtained worldwide after application of the OsseoFix System, the SpineJack System, radiofrequency kyphoplasty of the vertebral body, and the Kiva VCF treatment system. The following review discusses the development of the current techniques used for vertebral augmentation.

## 1. Introduction

Osteoporosis is characterized by decreased bone mass that leads to increased bone fragility and diminished structural support of the skeleton. The factors that lead to osteoporosis mainly include age, gender, lifestyle, drug effects, and autoimmune diseases, which disrupt the balance between osteogenesis and osteoclasts. Vertebral fractures secondary to osteoporosis are called osteoporotic vertebral fractures (OVCFs). One of the features that cause OVCFs is low energy damage. Because of the ascent of the ageing population, OVCFs, which are mainly caused by osteoporosis, have become one of the most major health problems worldwide. Approximately 20% of the elderly population is older than 70 years, and 16% of postmenopausal women worldwide experience OVCFs [1]. Furthermore, several complications of OVCFs, such as persistent pain, kyphotic deformity, weight loss, depression, reduced quality of life, and even death, have been reported [2]. Osteoporosis and its associated fractures are serious health issues in the ageing population. Indeed, vertebral compression fracture secondary to osteoporosis is a cause of morbidity and even mortality in older adults. Conservative therapies include bed rest, medications, bracing, physical therapy, exercise, and nerve root blocks. Conservative treatments are routinely used for OVCF patients; however, in cases of failed conservative treatment with insufficient pain relief after three weeks, vertebral augmentation should be considered. Moreover, it is inconclusive whether, based on current knowledge, conservative management is the best method for patients with OVCFs [3]. Conservative treatment, in addition, is ineffective in a large portion of patients [4]. Meanwhile, infectious diseases of the respiratory and urinary systems have been observed during the administration of conservative care, and hyperkyphosis is a common problem following OVCFs [5]. All of these elements have terrible impacts on patients with OVCFs. Since the introduction of minimally invasive surgery with its lower injury, shorter time, and rapid symptom relief, spinal surgeons, interventional radiologists, and others have become interested in vertebral augmentation techniques in recent years. These techniques mainly include percutaneous vertebroplasty (PVP), percutaneous kyphoplasty (PKP), and the SpineJack® System. The aim of this review is to analyse these devices that have been applied in recent years and to identify the differences among these new techniques.

Table 1 shows the comparison of several vertebral augmentation techniques, and Table 2 shows the summary of study characteristics [6–13]. Selection inclusion criteria and exclusion criteria: studies with the following criteria were included: (1) patients with osteoporotic lumbar and thoracic vertebra fractures; (2) random control trials or prospective or retrospective comparative studies; (3) moreover, studies which reported at least one of the following outcomes: vertebral height, cement leakage, adjacent vertebral fracture, visual analogue score, and Oswestry Disability Index. Studies were excluded in this article if they had neoplastic etiology, neurocompression, infection, traumatic fracture, neurologic deficit, spinal stenosis, severe degenerative diseases of spine, previous surgery at the involved vertebral body, and vertebral augmentation with other semi-invasive intervention treatments.

## 2. The Development of Vertebral Augmentation Systems

### 2.1. Percutaneous Vertebroplasty (PVP)

**Figure 1:**
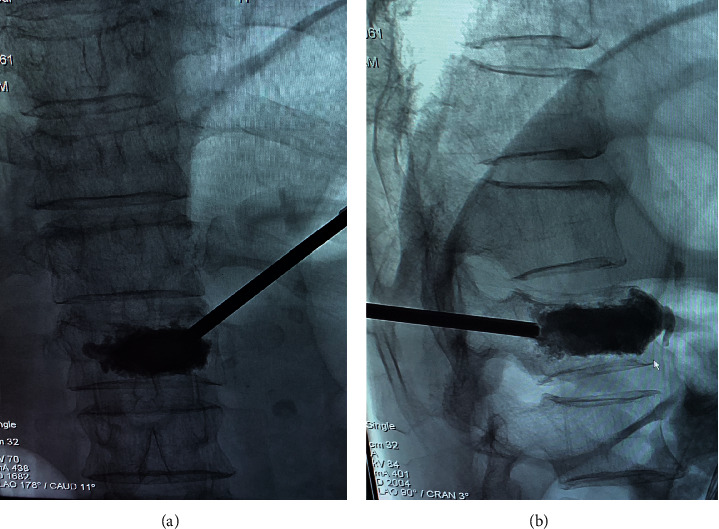
Male patient with back pain due to osteoporotic fracture of the L1 vertebral body. The frontal and the lateral fluoroscopy view—needles were placed in the anterior third of L1 vertebral bodies and cement injection was finished under continuous fluoroscopy.

In 1984, PVP was first introduced by Galibert and Deramond for treating haemangiomas at the C2 vertebra [14]. PVP has been used in patients with OVCFs who have failed conservative treatments to alleviate back pain and correct the deformity (Figure 1). The main predictors of favourable outcome among patients who have persistent and intense pain after OVCFs include early intervention and the absence of intravertebral clefts at 1 month after vertebroplasty [15].

Since its application in the treatment of OVCFs, various complications of PVP have been observed, such as neuraxial anaesthesia, severe cement embolism, new vertebral fractures, and infection after PVP. Cement leakage is one of the most common complications of this technique. The risk factors for cement leakage include the severity grade of the vertebral fracture, low viscosity of the polymethyl methacrylate bone cement, and the presence of intravertebral clefts; cortical disruption is also a risk factor for cement leakage [16–18]. One reason for cement leakage is early application of cement that has not reached its optimum viscosity. One of the efficient ways to detect cement leakage at an early stage is based on thorough fluoroscopic monitoring. The risk of cement leakage is approximately 30% since the cement extends beyond the confines of the bone because the low-viscosity cement is injected at a high pressure during the operation. Although the detected rate of cement leakage was found to be approximately 82% by using computed tomography (CT) [19], studies have indicated that most leakages are asymptomatic, among which, however, serious complications of nerve root or spinal cord compression and pulmonary embolism cannot be ruled out [16, 20, 21]. Moreover, adjacent vertebral fracture is one of the complications after PKP and cement leakage into the disc is considered the main factor increasing the risk of adjacent fracture [22].

The bipedicular approach was carried out as the standard technique of PVP. However, considering several aspects, such as operation time, cement volume, and radiation dose, a unipedicular approach was reported and advocated, as it reduced the operating time, limited X-ray exposure, and decreased the risk of cement leakage. The complications caused by vertebral pedicle puncture were decreased [23]. A meta-analysis conducted in 2016 indicated that there was no significant difference in the visual analogue score (VAS), the Oswestry Disability Index (ODI), or the rate of cement leakage. In addition, the operation time of unilateral PVP was shorter than that of bilateral PVP and this technique needed less cement [23]. Comparing the two surgery methods, these methods showed significant differences in pain relief, improvement of life quality, and radiological outcomes [23–25]. However, one study reported that the unipedicular approach might be associated with more nerve root stimulation [24].

PVP seems to be efficient and safe during the treatment of patients with OVCFs, and it can be performed at a reasonable cost with minimal complications [12]. For the time being, however, PVP should be cautiously considered for patients who have not yet received conservative therapy [26].

### 2.2. Percutaneous Kyphoplasty (PKP)

**Figure 2:**
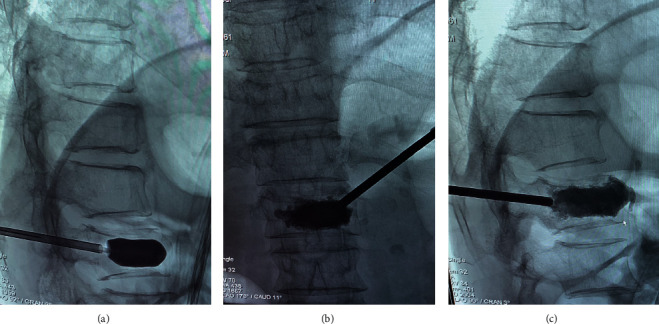
(a) The balloon was inflated to restore the height of the fractured vertebra and to create a cavity within the vertebra. (b) Frontal fluoroscopy view when bone cement was injected into the fractured vertebra. (c) Lateral fluoroscopy view when bone cement was injected into the fractured vertebra.

PKP is an improved technique based on PVP, which is applied to reduce the rate of bone cement leakage, better restore vertebral height, and stabilize the fractured vertebra at present. In addition, PKP is a safe and effective technique for the treatment of OVCFs (Figure 2). It was reported that compared with conservative medical care, balloon kyphoplasty significantly improved patient outcomes [27]. Furthermore, a randomized controlled trial (RCT) with a 24-month follow-up demonstrated that PKP relieved pain and improved motor function and quality of life more effectively than nonsurgical therapy without increasing the risk of additional vertebral fractures [28].

Both PKP and PVP are safe and effective surgical procedures for treating OVCFs [29]. However, in terms of restoring vertebral height and local kyphotic corrections, PKP is relatively better than PVP [30]. Studies of PKP have indicated that the procedure duration of PKP is short and this technique yields fewer cement leakages with better pain relief, improvements of ODI, and a trend towards a longer fracture-free survival [31, 32].

Although bone cement leakage is one of the most common complications of PKP as well, because balloon kyphoplasty forms a space in the fractured vertebra within the vertebral body, the bone cement can be injected under low pressure and the rate of bone cement leakage can be reduced to 1–8% [33]. However, the problem of bone cement leakage has not been completely solved. Although cement extravasation may lead to severe complications such as pulmonary cement embolism, PKP is superior to PVP because of the lower cement leakage rate [29]. Published research has indicated a cement leakage rate of approximately 9% in the PKP technique, while the cement leakage rate in PVP is as high as 41% [34]. Notably, in the treatment for bone cement leakage, no significant difference was found between PKP and PVP [35].

Both unilateral and bilateral PKP procedures show effectiveness for OVCFs. In addition, the unilateral puncture technique is reportedly superior to the bilateral puncture technique in several aspects: shorter operation time, lower radiation dose, and less injected bone cement [25]. One of the limitations of PKP, however, is that this technique produces significant displacement of the vertebra and damage to the trabeculae in the fractured vertebral body. Another problem is the loss of the fractured vertebral height between the period of removing the balloon and the period of injecting the cement. Moreover, studies have indicated that the influence of bone cement leakage caused by PKP is small and that there are no clinical symptoms among patients with cement extravasation [36, 37].

Both PVP and PKP increase bone strength as well as relieve pain caused by OVCFs, and both techniques rely on injecting polymethyl methacrylate (PMMA) cement into the fractured vertebra for mechanical stabilization of the OVCFs. Currently, mineralized collagen-modified PMMA (MC-modified PMMA), a kind of new bone cement that does not change the beneficial properties of PMMA, has better biocompatibility than normal PMMA. It has been reported that this new bone cement forms a stable structure in the vertebral body as well as improves the prognosis of patients who have OVCFs by reducing pain and reoperation [30]. With the development of biomaterials, it is possible to obtain new types of bone cement with bioactivity, excellent biomechanics, and even osteogenesis and appropriate degeneration.

### 2.3. OsseoFix® System

**Figure 3:**
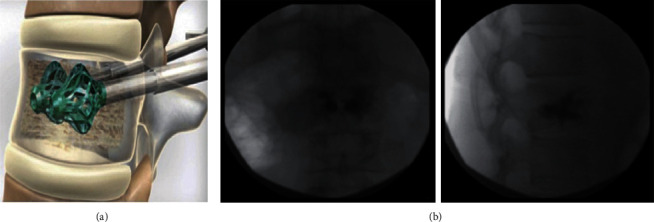
(a) OsseoFix® System. (b) Osteoporotic vertebral fracture lateral fluoroscopy view [39].

The OsseoFix® System (Alphatec Spine Inc., Carlsbad, California, USA) (Figure 3) is an expandable titanium mesh cage that is applied in the treatment of OVCFs and prevents kyphotic deformity by compacting the surrounding trabecular bone [38]. The cage is implanted into the anterior third of the vertebral body and then expanded slowly. The height of the fracture vertebra is restored because of the compaction of the trabecular bone by the titanium mesh cage. Subsequently, the cement is injected into the cage. Moreover, compared with the cement volume applied in PKP, significantly less cement is required in the utilization of the OsseoFix® System [39].

The OsseoFix® System has been available since 2009 and is a new percutaneous stabilization method for osteoporotic thoracolumbar vertebral compression fractures [40]. The OsseoFix® System has been applied for vertebral compression fractures among patients with T6 to L5 stable vertebral fractures (type A1.1 to A1.3 or A3.1, according to the Arbeitsgemeinschaft für Osteosynthesefragen (AO) classification). It has been reported that the OsseoFix® System is also useful in treating acute stable traumatic vertebral fractures of the same type among young patients. Moreover, the OsseoFix® System is well suited for stabilizing tumourous VCFs as well as osteoporotic VCFs. Several studies have indicated that vertebral fractures with intraspinal bone fragments, spinal cord compression, and previous treatment at the same level are the main contraindications for treatment with the OsseoFix® System [6, 39, 41].

A study of the clinical and radiological outcomes among patients with OVCFs showed that both the mean VAS (7.7–1.4) and mean ODI (70.6%–30.1%) showed significant improvements after treatment with the OsseoFix® System. Furthermore, according to the measurement of Cobb's angle, the mean kyphotic angle after the operation showed improvement (from 11.7° to 10.4° after 12 months). Meanwhile, despite one case of loss of height in a stabilized vertebral body (3.1%) [6], no complications, including adjacent vertebral fractures, were observed. The OsseoFix® System, which required less cement and provided significant height maintenance in vitro, was biomechanically similar to PKP [40]. It was suggested that the OsseoFix® System had an indirect mechanism of increasing vertebral body height and that the implant might be applied as a cement-free implant in future operations because of the special structure of the OsseoFix® System [6].

### 2.4. SpineJack® System

The SpineJack® System, a titanium implant, is mainly designed to restore the height of the vertebral body and is applied to treat OVCFs. It consists of a mechanical working system that allows controlled reduction of the vertebral fracture; the feature facilitates the recovery of collapsed vertebrae and provides 3D support to the structure, which is required to mechanically stabilize the vertebral body in axial compression [42]. After the reduction, PMMA is injected into the vertebral body to stabilize the reduction. This technique may now reduce the amount of cement injected, and this new augmentation method could also be a useful approach for treating traumatic fractures in young and middle-aged patients by using the combination of a permanent implant plus cement [43].

In a trial with an over 3-year follow-up, the results of percutaneous treatment performed with the SpineJack® System among patients with OVCFs indicated good long-term clinical efficiency and safety, especially in maintaining vertebral height and decreasing the risk of adjacent vertebral fracture; additional studies showed that compared with PKP, the SpineJack® System was more able to reduce mechanically compressed vertebral bodies and maintain height restoration than balloon kyphoplasty [44–49]. The bone cement volume was reduced to 10% with the SpineJack® System, while PKP required a 30% cement volume in the treatment of traumatic wedge fractures. It was reported that the SpineJack® System yielded positive function among patients with acute OVCFs. Furthermore, the treatment was performed after a mean delay of 5.8 months and showed that the effectiveness in correcting the structural damage was preserved in patients with chronic OVCFs [44–49].

Considering the short-term follow-up, the results and function of the SpineJack® system need to be studied in a larger series, and future studies should focus on long-term clinical and radiological outcomes.

### 2.5. Radiofrequency Kyphoplasty (RFK)

**Figure 4:**
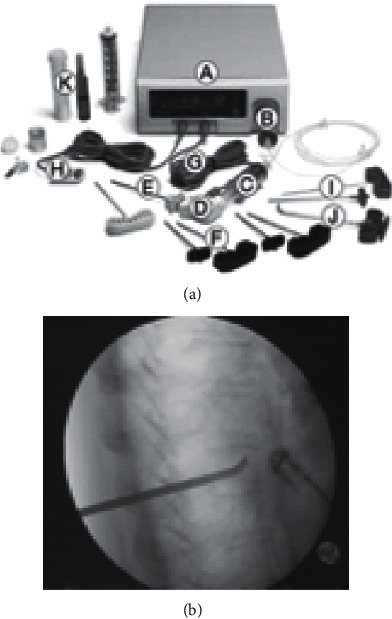
(a) Radiofrequency device and application system: (A) multiplex controller, (B) hydraulic assembly, (C) master syringe, (D) activation element, (E) locking delivery cannula, (F) StabiliT introducer-working cannula and stylet, (G) activation element cable, (H) hand switch cable, (I) straight line osteotome, (J) power curve navigating osteotome, and (K) StabiliT ER2 bone cement [52]. (b) Intraoperative X-ray of L1 vertebra (lateral view) using RFK [52].

Radiofrequency kyphoplasty, a kind of vertebral augmentation system, was introduced in Germany in 2009 with a unipedicular approach. With the help of an articulating osteotome, multiple channels are created within the cancellous bone of fractured vertebra, which preserves more intact cancellous bone than inflation of a balloon does (Figure 4) [50]. Then, ultrahigh viscosity cement is injected into the vertebral body. The procedure is accomplished by using the energy of radiofrequency to warm the cement and accelerate its polymerization.

The indications for RFK mainly include painful OVCFs in elderly patients (65 years of age) after conservative therapy failure, painful aggressive primary tumours of the spine, or osteolytic metastases to the spine with a high risk of vertebral fracture in the palliative care setting [51].

A study indicated that there was a significant reduction in VAS and that the improvement in ODI was approximately between 65% and 96%; furthermore, pain reduction and minimization of daily handicap were effectively achieved [11]. It was reported that RFK improved pulmonary function, especially when the fractures were in the main spinal region of the diaphragm [11]. Further study showed that FEV1 values improved after radiofrequency kyphoplasty. Thus, according to the inverse relationship between FEV1 and mortality risk, RFK may well reduce the risk of mortality [11]. In an in vitro study, compared with PKP, RFK achieved similar outcomes in both stabilizing and restoring the height of the fractured vertebra. In addition, the operational time was shorter and there was less damage to the trabecular bone [12]. RFK was effective for pain relief, and the risk of cement leakage was reduced. Moreover, in postoperative fractures and the secondary loss of high restoration, RFK performed better than PKP [8, 13].

However, more large-sample multicentred RCT studies are required in the future to validate this new surgical system.

### 2.6. Kiva VCF Treatment System

**Figure 5:**
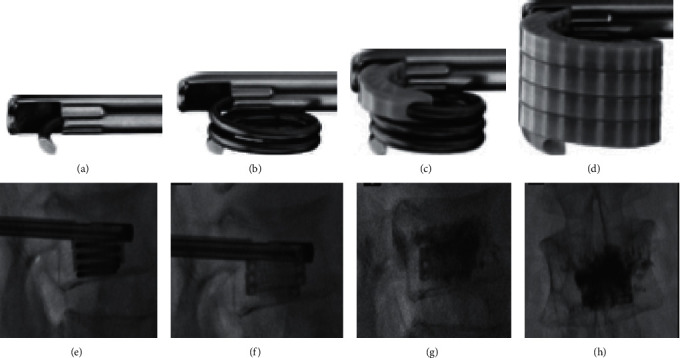
A percutaneous nitinol coil guidewire (a) is coiled within the cancellous portion of the fractured vertebral body. (b) Afterwards, a radiopaque PEEK implant is delivered incrementally via the nitinol coil guidewire (c) and then a nesting, cylindrical column is formed that provides vertical displacement, which may restore the height of the fractured vertebra (d) [10]. Fluoroscopic images illustrating the procedure of using the Kiva VCF treatment system (e). After removing the coil, a radiopaque PEEK implant was implanted (f) to provide structural support to the vertebral body and then bone cement was injected through the implant, as shown by lateral (g) and anteroposterior (h) fluoroscopic images [10].

The Kiva System is a novel alternative surgical equipment for treating OVCFs. In the procedure for utilizing this new technique, a nitinol Osteo Coil guidewire is advanced through a deployment cannula percutaneously. After correct placement of the nitinol coil in the cancellous portion, a polyether ether ketone (PEEK) implant (the implant contains 15% barium sulfate for radiopacity and forms a nesting, cylindrical column) is implanted incrementally over the coil until the desired restoration of the fractured vertebral height is achieved. Subsequently, the guidewire is removed and bone cement is injected through the pipe of the implant until the column is filled with cement (Figure 5).

The Kiva System was applied to patients with painful A1.1, A1.2, or A1.3 (AO spine fracture classification) OVCFs at the thoracic and lumbar spine or at an age at entry of 50 years or greater; 1–3 symptomatic OVCFs were considered [53]. Furthermore, a VAS score of 5 or greater, fracture age of less than 6 months, and an ODI score of 30% or greater were required [10].

The restoration of vertebral height could be maintained with both procedures for 6 months, and the Kiva group had fewer complications, such as adjacent fractures, than the PKP group had [53]. A previous study established that the rate of adjacent-level fracture with Kiva was reduced; therefore, the cost of treating OVCFs was reduced [2]. A study on the Kiva System showed that the mean back pain score on the VAS decreased by approximately 66% (*P* < 0.0001), and the improvement of the mean ODI score was approximately 63% at 12 months after operation. In addition, approximately 8% of cement extravasation was identified radiographically; however, no clinical symptoms were observed [10].

Compared with PKP, a study suggested that the Kiva System had identical outcomes, including the effective relief of pain. Kiva was shown to be noninferior to PKP and revealed a positive trend in several secondary endpoints [54]. Meanwhile, the Kiva System was found to be similar to PKP with respect to VAS and ODI, while less bone cement was needed via the Kiva System [2]. A comparison of the PKP and the Kiva System for OVCFs at 6 months after surgery indicated that the improvement in VAS in the Kiva group was significantly better than that in the PKP group (*P* < 0.0001), and the mean ODI scores in both groups also improved from 68.7% ± 15.8% to 24.8 ± 18.6% in the Kiva group and from 80.6% ± 8.6% to 33.2 ± 6.3% in the PKP group 6 months later. Furthermore, the mean operation time in the Kiva group was shorter than that in the PKP group, in which 12.7 ± 3.7 minutes per vertebra was observed in the Kiva System group and 34.1 ± 7.0 minutes per vertebra was observed in the PKP group [10].

The Kiva System can be effective for painful vertebral fractures [2, 10, 53, 55]. Longer observation, however, is needed to confirm whether the Kiva System provides positive functional improvement, and further randomized prospective studies with larger patient samples are necessary to predict long-term outcomes after the intervention [53, 56].

## 3. Conclusion

The principles of vertebral augmentation include improvements in functionality and back pain that promote the social life and independence of patients with OVCFs. Since not all vertebral compression fractures are the same, a tailored-based approach is necessary for optimum efficacy and safety [57]. Moreover, the surgical instruments, including balloons, the OsseoFix® System, the SpineJack® System, radiofrequency kyphoplasty, and the Kiva VCF system, have been improved. All of these techniques are utilized in clinics.

By comparing the outcomes of several vertebral augmentation techniques (Tables 1 and 2), these do have differences. According to our clinic experience, unilateral PKP has satisfied effects on vertebral augmentation, with less complications and medical cost. Although novel techniques have attractive effects on treatment of vertebral fracture, there is no clear indication that guides what kind of techniques we shall use. Besides, the outcomes of these novel techniques needed more clinical observation.

In addition, with the utilization and development of virtual reality (VR) and digital navigation in the field of spine surgery, the procedure and even the outcomes of the operation can be simulated in vitro. Before the real operation of vertebral augmentation, doctors can receive abundant training and practice in techniques such as finding the best angle and direction to inject bone cement. This approach could significantly shorten the operation time, reduce the pain of patients during the operation procedure, and avoid complications. Therefore, with the development of vertebral augmentation systems, the operation will be more efficient and safe. Moreover, with the application of novel theories, such as enhanced recovery after surgery (ERAS) and bone cement with compatible biomechanical properties and bioactivities, patients with OVCFs can achieve the maximum improvements in functionality as well as life ability and quality. Vertebral augmentation systems will likely undergo greater development than any other technical aspects.

## Figures and Tables

**Table 1:** The outcome comparison of different vertebral augmentation techniques.

	PVP		PKP		OS		SJS		RFK		KVT	
	Pre	Po	Pre	Po	Pre	Po	Pre	Po	Pre	Po	Pre	Po
MVH	8.5 ± 1.1	8.6 ± 1.1	8.6 ± 1.1	12.4 ± 2.8	8.3 ± 1.1	13.1 ± 1.8	8.4 ± 1.1	12.9 ± 1.8	8.3 ± 1.3	12.5 ± 1.4	8.4 ± 2.1	12.7 ± 1.6
KA	15.9 ± 5.5	11.3 ± 3.8	16.7 ± 7.8	8.8 ± 5.4	11.7	10.4	14.3	8.5	13.9	8.1	15.7	7.9
CL	—	20–70%	—	4–13.4%	—	4%	—	5.00%	—	6%	—	0.03%
AF	—	0–7.8%	—	25–26%	—	11.40%	—	12.50%	—	0–10%	—	13.8%
VAS	8.2 ± 1.8	4.1 ± 1.4	8.4 ± 1.0	3.8 ± 2.0	7.7	3.4	7.4 ± 1.3	4.1 ± 2.1	8.0 ± 1.1	3.5 ± 2.7	8.2 ± 1.5	3.9 ± 1.9
ODI	67.1 ± 16.2	36.8 ± 11.3	65.6 ± 15.8	36.4 ± 10.7	70.6%	30.6%	82.5%	25.7%	83.2%	23.6%	81.4%	24.5%

PVP, percutaneous vertebroplasty; PKP, percutaneous kyphoplasty; OS, OsseoFix® System; SJS, SpineJack® System; RFK, radiofrequency kyphoplasty; KVT, Kiva VCF Treatment System; MVH, middle vertebral height; KA, kyphotic angle; CL, cement leakage; AF, adjacent fracture; VAS, visual analogue score; ODI, Oswestry Disability Index; Pre, preoperative; Po, postoperative.

**Table 2:** Summary of study characteristics (population, gender, and etiology).

Techniques	Gender (*n*)		Mean age (year)	Fractured vertebral sites (*n*)
	Male	Female		
PVP	11	26	71.3 ± 10.0	T10–L5 (40)
PKP	10	17	64.6 ± 9.1	T4–L5 (32)
OS	5	9	75.2 ± 9.8	T11–L5 (15)
SJS	5	8	75.4 ± 8.4	T10–L5 (13)
RFK	3	6	75.3 ± 8.5	T12–L5 (11)
KVT	4	7	66.5 ± 9.1	T12–L5 (11)

PVP, percutaneous vertebroplasty; PKP, percutaneous kyphoplasty; OS, OsseoFix® System; SJS, SpineJack® System; RFK, radiofrequency kyphoplasty; KVT, Kiva VCF Treatment System.
